# A stepwise loading method to magnetically responsive Pt-Fe_3_O_4_/MCNT catalysts for selective hydrogenation of 3-methylcrotonaldehyde

**DOI:** 10.1186/1556-276X-9-677

**Published:** 2014-12-13

**Authors:** Shaofei Song, Jianyan Yu, Qiang Xiao, Xiangrong Ye, Yijun Zhong, Weidong Zhu

**Affiliations:** Key Laboratory of the Ministry of Education for Advanced Catalysis Materials, Institute of Physical Chemistry, Zhejiang Normal University, Jinhua, 321004 People’s Republic of China

**Keywords:** Magnetic, Platinum, Hydrogenation, Unsaturated aldehyde

## Abstract

Pt-loaded multi-walled carbon nanotubes (Pt/MCNTs) and magnetically responsive Pt-Fe_3_O_4_/MCNT catalysts were prepared by a stepwise loading of preformed Pt and Fe_3_O_4_ nanoparticles onto multi-walled carbon nanotubes (MCNTs). The structure, composition, and magnetism of the catalysts were characterized by X-ray diffraction (XRD), TEM, H_2_-O_2_ titration, inductively coupling plasma-atomic emission spectroscopy (ICP-AES), and superconducting quantum interference device (SQUID) techniques. Ascribed to the well-controlled particle size in the preformed Pt colloids, Pt particles in the consequent Pt/MCNT and Pt-Fe_3_O_4_/MCNT catalysts are of high uniformity and dispersion. The prepared Pt catalysts show an excellent catalytic performance in the liquid phase hydrogenation of 3-methylcrotonaldehyde, one of typical α,β-unsaturated aldehydes. A very high selectivity to 3-methylcrotonalcohol of 98% at a conversion of about 80% was available on the magnetic Pt-Fe_3_O_4_/MCNT catalyst. The magnetic catalyst, with good superparamagnetism, can be easily recovered from the liquid phase system under the external magnetic field. Moreover, both the Pt/MCNT and magnetic Pt-Fe_3_O_4_/MCNT catalysts show a good recyclability, confirmed by five cycles of reusage.

**Electronic supplementary material:**

The online version of this article (doi:10.1186/1556-276X-9-677) contains supplementary material, which is available to authorized users.

## Background

**Figure 1:**
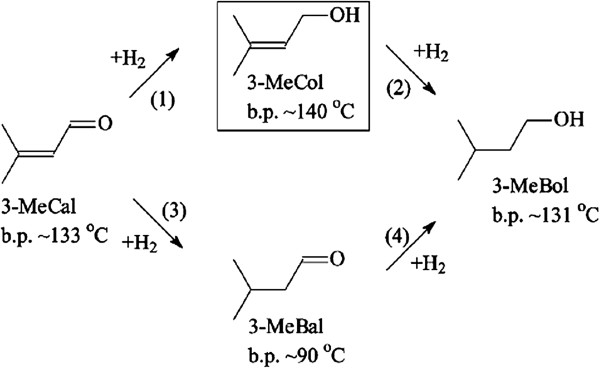
**Hydrogenation pathway of 3-MeCal where the desired product 3-MeCol is encircled.**

Selective hydrogenation of α,β-unsaturated aldehyde is an important reaction in industry for the synthesis of fine chemicals. As illustrated in Figure 1, the hydrogenation of 3-methylcrotonaldehyde (3-MeCal), one of typical α,β-unsaturated aldehydes, leads to 3-methylcrotonalcohol (3-MeCol), 3-methylbutyraldehyde (3-MeBal), and 3-methyl-1-butanol (3-MeBol) as the main products. The half-hydrogenated C = O product 3-MeCol as an intermediate finds wide application in the production of perfumes and pesticides. However, the hydrogenation of the C = C bond is more favorable based on both thermodynamic and kinetic considerations [1]. Additionally, due to the very close boiling points (3-MeBol: approximately 131°C and 3-MeCal: approximately 133°C), it is rather difficult to separate the fully hydrogenated product 3-MeBol from the starting material 3-MeCal by distillation. Consequently, the selective hydrogenation of 3-MeCal into 3-MeCol is highly desirable yet of a great challenge [2, 3].

Metallic catalysts, e.g., Pt, Pd, Ru, Cu, Co, and Au, have been intensively studied in the selective hydrogenation of α,β-unsaturated aldehydes [4–10]. Among the most active and selective catalysts, Pt has been retained in the majority of the published work for the selective hydrogenation of α,β-unsaturated aldehydes [11–14]. Its selectivity depends critically on the particle size, i.e., the larger the particle size, the higher the selectivity to the unsaturated alcohol [15–18]. Smaller Pt particles would reduce the selectivity to unsaturated alcohol. As a result, heterogeneity in size distribution would induce the descent of the overall selectivity. It is therefore highly desirable to have a narrow-sized distribution of Pt nanoparticles which exhibits decent selectivity in addition to high conversion. Loading a preprepared Pt colloid on support has been practiced to prepare supported Pt particles with uniform size [19]. However, organic stabilizers are often used in the preparation of colloidal Pt solution. After the Pt particle deposition on supports, the stabilizer has to be removed typically by heat treatment, during which, unfortunately, the size of Pt particles varies, depending on the temperature and time applied in heat treatment.

On the other hand, the filtration-based recovery of expensive Pt catalyst from the liquid reaction system is rather time- and energy-consuming. The introduction of magnetically responsive substances to the catalyst could endow the catalyst magnetic properties, which makes it easy to recover the catalyst under a magnetic field and improves the separation efficiency [20–24]. Although magnetically responsive catalyst has been intensively reported [25–31], scarce work has been dedicated to their application in the selective hydrogenation of unsaturated aldehydes [32].

**Figure 2:**
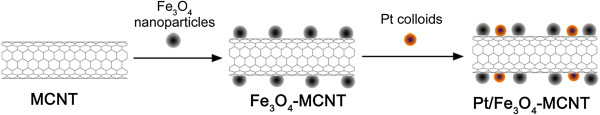
**Preparation process of a stepwise loading method to magnetic Pt-Fe**_**3**_ **O**_**4**_ **/MCNT catalyst.**

In this paper, we adopt a stepwise loading approach using multi-walled carbon nanotube (MCNT) as a model support to prepare Pt-loaded multi-walled carbon nanotube (Pt/MCNT) and magnetically responsive Pt-Fe_3_O_4_/MCNT catalysts. As illustrated in Figure 2, the uniform-sized Pt and Fe_3_O_4_ nanoparticles were beforehand prepared by a two-phase liquid-liquid method and a simple organic-phase method, respectively. Then the Fe_3_O_4_ and Pt nanoparticles were successively dispersed on MCNT. Ascribed to the high uniformity in the size of Pt and Fe_3_O_4_ nanoparticles, a good performance in the selective hydrogenation of 3-MeCal in terms of selectivity, recovery, and reusage is highly anticipated.

## Methods

### Catalyst preparation

#### Pt/MCNT

The Pt nanoparticles were prepared by a two-phase liquid-liquid method (for more details, see Additional file 1). The as-made Pt nanoparticles were dispersed in toluene (15 mL) and then mixed with an appropriate aliquot of MCNT. After ultrasonic treatment for 1 h, the mixture was magnetically stirred overnight. Finally, Pt/MCNT catalyst was recovered by filtration and dried at 80°C overnight. The Pt/MCNT catalysts are referred to as *x*Pt/MCNT, where *x* is the stoichiometric loading in weight percent.

#### Pt-Fe_3_O_4_/MCNT

Fe_3_O_4_ nanoparticles were prepared by a simple organic-phase synthesis (for more details, see Additional file 1) [33, 34]. The prepared Fe_3_O_4_ nanoparticles were redispersed in hexane in the presence of oleic acid and oleylamine and mixed with an appropriate aliquot of MCNT. The mixture was ultrasonicated for 1 h and stirred overnight. Then the magnetic composites were recovered by filtration and dried at 100°C and referred to as *y*Fe_3_O_4_/MCNT, where *y* is the stoichiometric loading in weight percent. The as-made Pt nanoparticles were redispersed in toluene (15 mL) and then the as-made Fe_3_O_4_/MCNT magnetic composite was added. After ultrasonic treatment for 1 h and magnetically stirred overnight, the mixture was filtered and dried at 80°C overnight to obtain *z*Pt-*y*Fe_3_O_4_/MCNT magnetic catalysts. Here, *z* is the stoichiometric loading in weight percent based on MCNT.

### Catalyst characterization

X-ray diffraction (XRD) patterns were recorded on a Philips PW3040/60 diffractometer (Philips Analytical, Almelo, Netherlands) using CuKα radiation (*λ* = 1.54 Å). The scans were recorded in the 2θ range between 10° and 80° with a step width of 0.03°. Transmission electron microscopy (TEM) observations were carried out on a 2100 JEOL TEM (JEOL Ltd., Akishima, Tokyo, Japan) working at 200 kV. The sample was diluted in ethanol to give a 1:5 volume ratio and sonicated for 10 min. The ethanol slurry was then dripped onto a Cu grid covered with a holey film of carbon. H_2_-O_2_ titration measurements were performed on a Micromeritics AutoChem II chemisorption analyzer (Micromeritics Instrument Co., Norcross, GA, USA) to determine the Pt dispersion of the Pt/MCNT catalysts. Prior to the measurement, the catalyst was preheated in a helium flow at 373 K for 30 min and completely reduced in an Ar flow with 10 vol.% H_2_ at 573 K for 1 h. The concentrations of the effluent gases were monitored by a calibrated thermal conductivity detector (TCD). Pt loading of the Pt/MCNT and Pt-Fe_3_O_4_/MCNT catalyst was determined by a Jarrell-Ash 1100 inductively coupling plasma-atomic emission spectrometer (ICP-AES, MOA Instrumentation, Inc., Levittown, PA, USA). Each sample was analyzed three times and the results were averaged. The magnetic measurements were carried out on a superconducting quantum interference device (SQUID, MPMS, Quantum Design, San Diego, CA, USA) at 27°C. About 2.5 g of sample was used in each measurement.

### Hydrogenation reaction

Hydrogenation of 3-MeCal was carried out in a 50-mL Teflon-lined stainless steel autoclave equipped with a hydrogen inlet, a pressure gauge, and a thermocouple. Prior to the first reaction, the catalyst was activated in a N_2_ flow at 300°C for 1 h. The catalyst (0.1 g), 3-MeCal (2.0 mL), deionized water (2.0 mL), and ethanol (16.0 mL) were mixed and sealed into the autoclave under vigorous magnetic stirring at a speed of 960 rpm. For the sake of safety, N_2_ was firstly charged to replace the air, and subsequently, H_2_ was charged several times. Afterwards, the autoclave was heated up to 80°C. The reaction was started by raising H_2_ pressure up to 1.4 MPa. The reaction solution was periodically sampled and analyzed by a gas chromatography Shimadzu GC-2014 (Shimadzu, Kyoto, Japan) equipped with an FID detector and a DB-5 capillary column. As for the recovery of Pt-Fe_3_O_4_/MCNT, a magnet with a magnetic field strength of 10.5 to 11.0 kOe was put near the Teflon container, driving the magnetic catalyst to be attached to the side with the magnet. Then, the liquid phase was poured out. The catalyst was washed with ethanol twice and used for a next reaction without activation.

## Results and discussion

### Pt/MCNT

**Figure 3:**
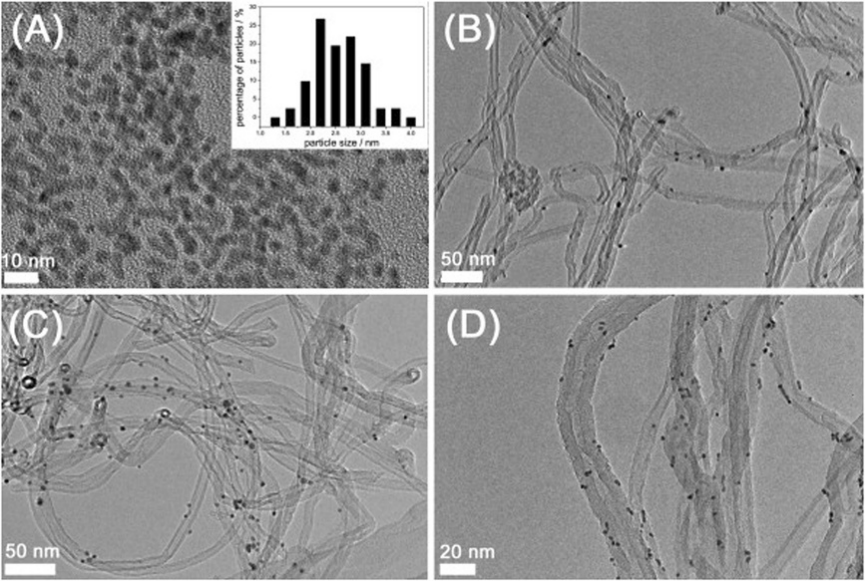
**TEM images of the as-prepared Pt colloids and particle size distribution histogram (A), 0.5Pt/MCNT (B), 3Pt/MCNT (C), and 5Pt/MCNT (D).**

A two-phase (water-toluene) reduction method was adopted to prepare uniform-sized Pt colloids with TOAB and oleylamine as stabilizers. Figure 3A shows the TEM image of the as-obtained Pt colloids, suggesting the monodispersion and good uniformity of the Pt nanoparticles. The corresponding histogram obtained from the TEM image indicates a narrow Pt particle size distribution ranging from 1.5 to 3.5 nm. The weighted average particle size is determined to be 2.6 nm (see Additional file 1: Equation 1). Pt/MCNT catalysts with different Pt loadings were prepared by direct loading the Pt particle colloids on MCNTs followed by an identical activation procedure. TEM images show that Pt nanoparticles are well dispersed on the outer surface of MCNTs without altering the particle size (Figure 3B,C,D).

**Figure 4:**
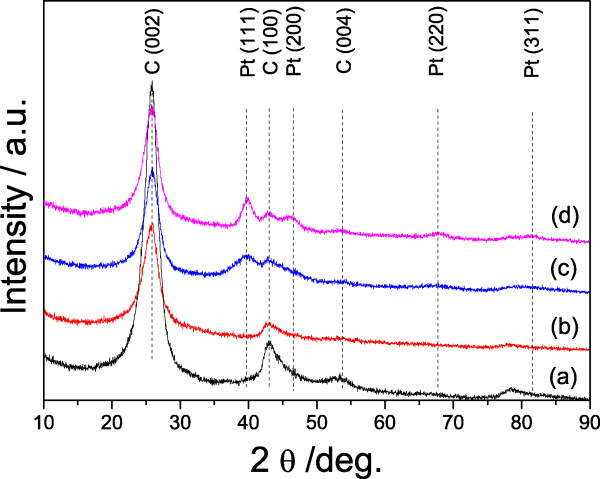
**XRD patterns of the MCNT (a), 0.5Pt/MCNT (b), 3Pt/MCNT (c), and 5Pt/MCNT (d).**

The XRD patterns of the Pt/MCNT catalysts are presented in Figure 4 alongside the pristine MCNT used as a comparison. As shown in Figure 4(a), the pattern for MCNT exhibits typical diffraction peaks at 25.9°, 43.2°, and 53.9°, which can be assigned to diffraction from the (002), (100), and (004) planes, in agreement with those for MCNTs [35, 36]. Besides the peaks assigned to MCNT phase, a diffraction peak of Pt (111) plane is observed for the Pt/MCNT catalysts. With the increase of Pt loadings, the intensity of the Pt (111) peak increases accordingly.

The as-prepared Pt/MCNT catalysts exhibit good performance in the hydrogenation (see Additional file 1: Figure S1). On the three catalysts, the molar fraction of 3-MeCol in the reaction system linearly increases with the reaction going on, and more than 95% selectivity to 3-MeCol can be achieved at the initial reaction period. The initial reaction rate increases with increasing of the Pt loadings. Selectivities higher than 92% are always available even at the conversions more than 95%.

Initial turn-over frequencies (TOFs) and dispersions of the Pt/MCNT catalysts are reported in Table 1. The as-prepared Pt/MCNT catalysts show almost the same Pt particle sizes determined by either the TEM technique or the H_2_-O_2_ titration. The calculated initial TOFs show approximately the same values for the three catalysts, accounting for the similar activity of the Pt particles on Pt/MCNT catalysts whatever the Pt loadings. The similar TOF values indicate that the Pt particles maintain their original uniform sizes during the preparation process.

**Table 1:** **Pt particle data of the Pt/MCNT catalysts and TOF values in the hydrogenation of 3-methylcrotonaldehyde (3-MeCal)**

Samples	Pt loading/wt.%	Pt particle size (nm)		Dispersion (%)	initial TOF ^c^ (s ^-1^ )
		TEM ^a^	H _2_ -O _2_ titration ^b^		
0.5Pt/MCNT	0.5	2.6	2.7	41.6	1.8
3Pt/MCNT	2.9	2.6	2.8	40.5	1.5
5Pt/MCNT	4.8	2.6	2.9	39.4	1.3

^a^The average value determined by TEM observations; ^b^determined by the H_2_-O_2_ titration; ^c^calculated from the data in Additional file 1: Figure S1.

The catalyst was recovered through filtration after reaction, washed with ethanol twice, and then used for the next reaction without other treatments. The 3Pt/MCNT catalyst, as an example, maintained its original activity and selectivity during the five cycles (Additional file 1: Table S1). The Pt loading was monitored by inductively coupling plasma (ICP), showing that no leaching of Pt occurs.

### Magnetic Pt-Fe_3_O_4_/MCNT catalysts

The magnetic phase of Fe_3_O_4_ was introduced to the catalyst by the stepwise loading method to prepare the magnetic responsive catalyst. Easy catalyst recovery from the reaction system is anticipated under the magnetic field.

**Figure 5:**
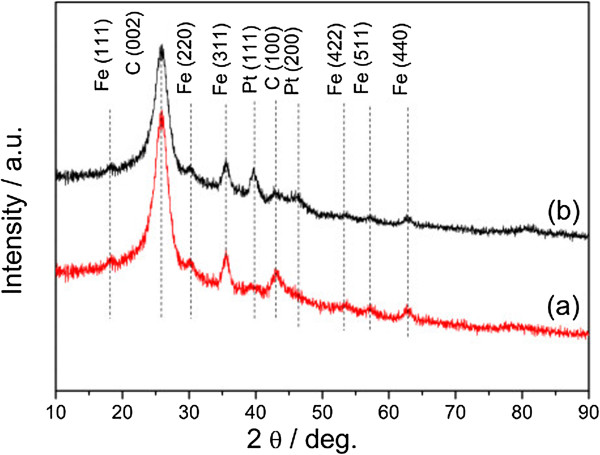
**XRD patterns of (a) 5Fe**_**3**_ **O**_**4**_ **/MCNT and (b) 3Pt-5Fe**_**3**_ **O**_**4**_ **/MCNT.**

Diffraction peaks at 18.3°, 30.1°, 35.4°, 53.6°, 56.9°, and 62.5°, which can be attributed to (111), (220), (311), (422), (511), and (440) planes of Fe_3_O_4_ phase, respectively, can be observed for the Fe_3_O_4_/MCNT composite, indicating that the Fe_3_O_4_ phase has been successfully supported on MCNTs (Figure 5). Additionally, diffraction peak assigned to Pt (111) also appears besides the peaks attributed to Fe_3_O_4_ and MCNT (Figure 5).

**Figure 6:**
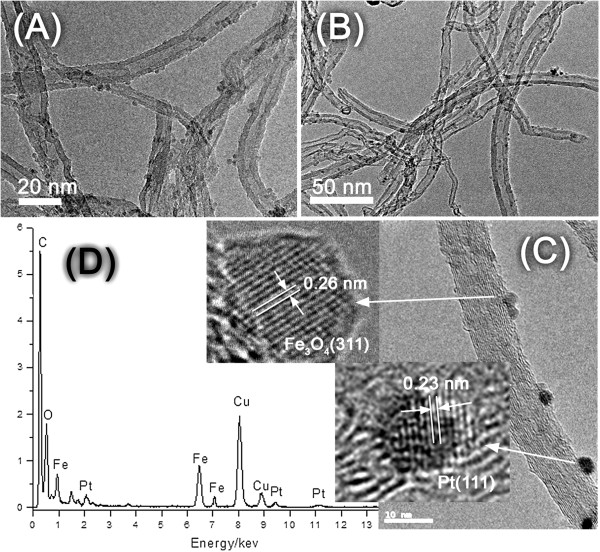
**TEM images of (A) 5Fe**_**3**_ **O**_**4**_ **/MCNT, (B, C) 3Pt-5Fe**_**3**_ **O**_**4**_ **/MCNT, and (D) EDS spectrum of 3Pt-5Fe**_**3**_ **O**_**4**_ **/MCNT.**

The TEM image of the magnetic composite (5Fe_3_O_4_/MCNT) reveals that Fe_3_O_4_ nanoparticles with diameter of about 2 to 4 nm are well dispersed on the outer surface of MCNTs (Figure 6A). While on the 3Pt-5Fe_3_O_4_/MCNT catalyst, some dark spots attributed to the Pt nanoparticles appear besides the grey spots of Fe_3_O_4_ nanoparticles (Figure 6B). From the magnified image (Figure 6C), one can see the highly dispersed Pt and Fe_3_O_4_ nanoparticles separately located on the surface. The high resolution TEM image shows that the nanoparticles are single crystalline, confirmed by atomic lattice fringes (Figure 6C, inset). For a grey spot, the distance between two planes is about 0.26 nm, in agreement with Fe_3_O_4_ (311) plane. While, for a dark spot, lattice spacing is about 0.23 nm, which can be attributed to the Pt (111) plane. Additionally, from the energy-dispersive X-ray spectroscopy (EDS), characteristic X-rays assigned to Fe and Pt elements can be observed, further confirming that Pt and Fe_3_O_4_ nanoparticles have been successfully supported on MCNT (Figure 6D).

**Figure 7:**
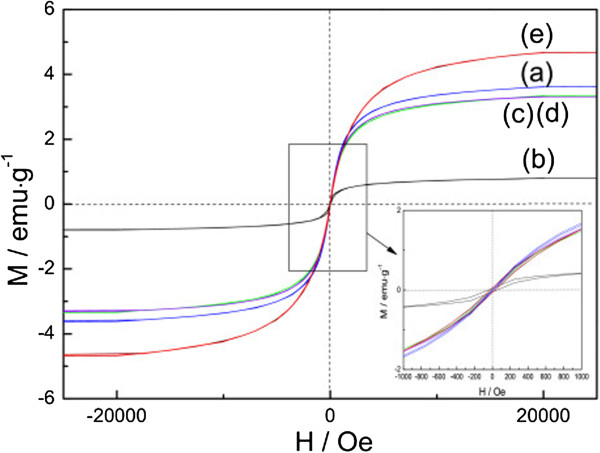
**Magnetization curves of (a) 5Fe**_**3**_ **O**_**4**_ **/MCNT, (b) 3Pt-1Fe**_**3**_ **O**_**4**_ **/MCNT, (c) 3Pt-5Fe**_**3**_ **O**_**4**_ **/MCNT, (d) five-cycles-used 3Pt-5Fe**_**3**_ **O**_**4**_ **/MCNT, and (e) 3Pt-10Fe**_**3**_ **O**_**4**_ **/MCNT.** Inset: the magnification of the square circle.

The magnetic properties of Fe_3_O_4_/MCNT magnetic composite and Pt-Fe_3_O_4_/MCNT magnetic catalysts were investigated by a magnetic property measurement system (MPMS) at room temperature. As shown in Figure 7, all the hysteresis loops go through the origin. Almost no remanence exists when the external magnetic field is removed (Figure 7, inset), revealing the superparamagnetism of the samples. The superparamagnetism implies that the magnetic responsive catalysts can be well redispersed in the absence of a magnetic field. The saturated magnetization value of 5Fe_3_O_4_/MCNT is about 3.6 emu·g^-1^. While, for 3Pt-5Fe_3_O_4_/MCNT, the value drops to 3.3 emu·g^-1^. Such a drop may be attributed to the decrease of the relative amount of Fe_3_O_4_ after loading with Pt. The saturated magnetization values of 3Pt-1Fe_3_O_4_/MCNT, 3Pt-5Fe_3_O_4_/MCNT, and 3Pt-10Fe_3_O_4_/MCNT are 0.8, 3.3, and 4.7 emu·g^-1^, respectively. The magnetization of these magnetic samples enhances with the increase of the Fe_3_O_4_ loading. The saturated magnetizations of Fe_3_O_4_ nanoparticles calculated from either Fe_3_O_4_/MCNT or Pt-Fe_3_O_4_/MCNT are 77 to 81 emu·g^-1^, in accordance with the values reported by Sun et al. [33]. Moreover, the magnetization curves coincide very well for the fresh- and five-cycles-used 3Pt-5Fe_3_O_4_/MCNT, indicating the good stability in magnetism.

**Figure 8:**
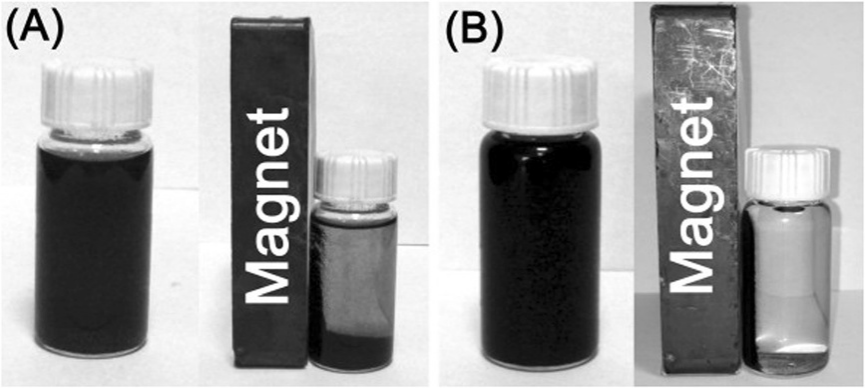
**Photographs of the samples dispersed in ethanol before and after the separation by magnet. (A)** 3Pt/1Fe_3_O_4_-MCNT, **(B)** 3Pt/5Fe_3_O_4_-MCNT.

The catalysts were dispersed in ethanol (solvent of hydrogenation) and formed black suspensions. After the catalysts were put in the external magnetic field for 5 min, they were attracted close to the side near the magnet and thus separated from the solvent. For 3Pt-1Fe_3_O_4_/MCNT, the separation is inefficient and part of the catalyst sinks to the bottom. By contrast, the magnetism of the 3Pt-5Fe_3_O_4_/MCNT catalyst is strong enough to be separated completely from the solution with the help of an external magnetic field (Figure 8 and Additional file 2).

The selective hydrogenation of 3-MeCal was performed over the prepared magnetic catalysts, and the results are collected in Table 2. The hydrogenation over the bare 5Fe_3_O_4_/MCNT magnetic composite was investigated as reference and no conversion was observed, implying the inertness of sole Fe_3_O_4_ in the hydrogenation. By contrast, the 3Pt/MCNT catalyst performs good properties in the hydrogenation. The introduction of magnetic responsive phase reduces the conversion of 3-MeCal. With the increase of the Fe_3_O_4_ loading amount, the conversion on the prepared Pt-Fe_3_O_4_/MCNT decreases correspondingly, due to the decrease of the relative Pt loading. The catalytic activity was normalized by the Pt loading and expressed as specific reaction rate (SRR). The SRR values are very close for all the Pt catalysts (Table 2). On the other hand, a slight increase of selectivity to the desired product of 3-MeCol can be observed at a higher Fe_3_O_4_ loading amount. A selectivity as high as 99.3% is available on the 3Pt-10Fe_3_O_4_/MCNT at a conversion of approximately 60 %. Since scarce work has been dedicated to the liquid hydrogenation of 3-MeCal, limited reference data related to the conversion and selectivity can be obtained. However, such a selectivity of more than 99% at a conversion up to 60% is of a high level among data from opening literatures related to the hydrogenation of cinnamaldehyde [1].

**Table 2:** **Results of the hydrogenation of 3-MeCal over Pt-Fe**_**3**_ **O**_**4**_ **/MCNT catalysts**

Catalysts	Pt loading (wt.%)	Fe _3_ O _4_ loading (wt.%)	***Conversion*** (%)	Specific reaction rate (10 ^- 2^ mol·g ^- 1^ ·min ^- 1^ )	Selectivity (%)		
					3-MeCol	3-MeBal	3-MeBol
5Fe_3_O_4_-MCNT	-	4.4	0	-	-	-	-
3Pt/MCNT	2.9	-	88.3	1.77	97.0	0.6	2.4
3Pt/1Fe_3_O_4_-MCNT	2.9	1	85.9	1.72	97.6	0.6	1.8
3Pt/5Fe_3_O_4_-MCNT	2.7	4.3	79.7	1.71	98.8	0.3	0.8
3Pt/10Fe_3_O_4_-MCNT	2.3	6.1	59.4	1.50	99.3	0.3	0.4

Reaction conditions: T = 80°C, t = 3 h; catalyst amount: 0.1 g; 3-MeCal dose: 2 ml; ethanol dose: 16 ml; deionized water: 2 ml; initial H_2_ pressure: 1.4 MPa. No H_2_ was fed during the reaction.

Hydrogenation of 3-MeCol over the 3Pt-5Fe_3_O_4_/MCNT was conducted to investigate the hydrogenation of the C = C bond (see Additional file 1: Figure S2). It can be seen that the conversion of 3-MeCol over 3Pt-5Fe_3_O_4_/MCNT is only 1.0% even after the reaction for 8 h under the same reaction conditions. The non-preferential hydrogenation of C = C over the catalyst leads to the very high selectivity to 3-MeCol.

3Pt-5Fe_3_O_4_/MCNT catalyst, as an example, can be facilely separated from the liquid reaction system with the help of an external magnetic field. During five cycles, the magnetic responsive catalyst maintains a stable performance in terms of both conversion of 3-MeCal and selectivity to 3-MeCol, which is about 80% and 98%, respectively, displaying a good recyclability (Additional file 1: Table S2). The Pt loading of the catalyst after being used for five times was checked keeping about 2.7 wt.%, and no Pt was detected in the reaction solution by ICP technique, indicating that no Pt leaching happens during the hydrogenation.

## Conclusions

Pt/MCNT catalysts were prepared by loading the forehanded Pt nanoparticles onto MCNT. Monodispersed Pt particles with high uniformity in size are available during the preparation of Pt colloids. Thus, the Pt particles on the consequent Pt/MCNT catalysts are of good uniformity with size around 2.6 nm and of high dispersion (up to 40%), resulting in an excellent catalytic performance in the liquid phase hydrogenation of 3-methylcrotonaldehyde. Subsequently, Pt-Fe_3_O_4_/MCNT magnetic catalysts were prepared by adopting the developed stepwise loading method. The prepared magnetic responsive catalysts exhibit good superparamagnetic behaviors and can be facilely separated from the liquid solvent. The hydrogenation of 3-MeCal results reveal that the magnetic catalysts show excellent hydrogenation properties, achieving a high selectivity to 3-MeCol of 98% at a conversion of more than 80%. Additionally, the magnetic responsive catalysts show a good recyclability. No decay of magnetic and catalytic properties was observed after five-cycle usage.

## Electronic supplementary material

Additional file 1: **Experimental details, hydrogenation results, and recycling tests on the catalysts.** (DOC 214 KB)

Additional file 2: **Movie of a magnetically driven separation process of the 3Pt-5Fe**_**3**_ **O**_**4**_ **/MCNT catalyst from the suspension.** (AVI 2 MB)
