# Supplementary material for: A stepwise loading method to magnetically responsive Pt-Fe3O4/MCNT catalysts for selective hydrogenation of 3-methylcrotonaldehyde
Source: Nanoscale Res Lett. 2014 Dec 13;9:677. doi: 10.1186/1556-276X-9-677 (PMC4493847; doi:10.1186/1556-276X-9-677)
Supplement: Supplementary file 1 — Additional file 1: Experimental details, hydrogenation results, and recycling tests on the catalysts. (DOC 214 KB) [file 11671_2014_2498_MOESM1_ESM.doc]

Additional File 1

# Magnetically responsive Pt-Fe_3_O_4_/MCNT catalysts with extraordinary C=O selectivity in hydrogenation of 3-methylcrotonaldehyde

Shaofei Song, Jianyan Yu, Qiang Xiao*, Xiangrong Ye*,Yijun Zhong, Weidong Zhu

Key Laboratory of the Ministry of Education for Advanced Catalysis Materials, Institute of Physical Chemistry, Zhejiang Normal University, Jinhua, 321004 P. R. China

Tel: +86 579 82283457; Fax: +86 579 82282234;

E-mail: [xiaoq@zjnu.edu.cn](mailto:xiaoq@zjnu.edu.cn)

E-mail: [yxr@zjnu.cn](mailto:yxr@zjnu.cn)

Experimental in detail

**Materials**

Multi-walled carbon nanotubes (MCNTs) with BET surface area and pore volume of 202 m^2^/g and 0.46 cm^3^/g, respectively, were obtained from CNano Technology Ltd., Beijing, China. H_2_PtCl_6_·6H_2_O was purchased from Xi'An Catalyst Chemical Co. Ltd., Xi'An, China. NaBH_4_ (purity >96%) was purchased from Shanghai Lianshi Chemicals Co., Ltd., Shanghai, China. Tetraoctylammonium bromide (TOAB, purity ~98%), oleylamine (purity: 80~90%), oleic acid (purity: 99%), Diphenyl ether (purity~99%) were purchased from Aladdin Reagent Co., Ltd. Toluene (purity> 98%) and n-butane (purity> 98%) were purchased from Quzhou Juhua Reagent Co., Ltd. Iron(III) acetylacetonate [Fe(acac)_3_, purity: 97 %], diphenyl ether (DPE, purity: 99%), 1,2-Hexadecanediol (HDD, purity: 90%) and 3-MeCal (purity >97 %) were purchased from Sigma-Aldrich Co. LLC.

**Preparation of Pt nanoparticles**

The Pt nanoparticles were prepared by a two-phase liquid-liquid method.[^1^](#_ENREF_1) In a typical synthesis, TOAB (0.1 g) and H_2_PtCl_6_ solution (5 mL, 0.1 g/mL) were added to toluene (20 mL). The mixed solution was stirred for 2 h until the aqueous phase became colorless. Then oleylamine (0.061 mL) was added to the above solution. After stirring for 1 h, fresh prepared NaBH_4_ (5 mL, 7.4 g/L) was added and stirred for another 2 h. Dark brown solution containing the Pt colloids was formed in this manner. Finally, ethanol (100 mL) was added to the above solution and then centrifuged at 3500 r/min for 10 min to collect the Pt nanoparticles. The Pt nanoparticles were washed with ethanol and distilled water by repeated centrifugation and decanting of the supernatant.

**Preparation of** **Fe_3_O_4_ nanoparticles**

Fe_3_O_4_ nanoparticles were prepared by a simple organic-phase synthesis. In a typical synthesis, Fe(acac)_3_ (0.71 g, 2 mmol), HDD (2.58 g, 10 mmol), oleylamine (1.61 g, 6 mmol), and oleic acid (1.69 g, 6 mmol) were added to of DPE (20 mL) in sequence under nitrogen and ultrasonicated for 30 min. Then the solution was heated to reflux for 1 h to form a dark-brown mixture. After cooled to room temperature, ethanol (40 mL) was added to the dark-brown mixture under air, and a dark-brown precipitate was collected by repeated centrifugation.

**Pt particle size determined from TEM**

The Pt particle size distribution were determined by measuring 100-200 particles from the TEM images. The weighted average size was calculated by the following equation:

$d_{av}=\sum_{i=1}^{n} n_{i}d_{i}^{2}/\sum_{i=1}^{n} n_{i}d_{i}$ (1)

where *d_av_* is the average diameter of the Pt particles, *d_i_* is the diameter of the determined Pt particle, and *n_i_* is the total number of the measured Pt particles.

**Pt particle size determined from H_2_-O_2_ titration measurement**

H_2_-O_2_ titration measurement was performed on Micromeritics AutoChem II chemisorption analyzer to determine the Pt dispersion of the Pt/MCNT catalysts. Prior to the measurement, the catalyst was pre-heated in a flowing helium at 373 K for 30 min and completely reduced in an Ar flow with 10 vol.% H_2_ at 573 K for 1 h. The concentrations of the effluent gases were monitored by a calibrated thermal conductivity detector (TCD). It is assumed that one H_2_ molecule can be split into two H atoms that occupy two Pt active sites. The average particle size was used to calculate the dispersion of Pt on the catalyst using the formula described by Scholten et al.,[^4^](#_ENREF_4) assuming that the Pt particles are spherical in shape, as follows:

(2)

where *D* is the dispersion (Pt*_surface_*/Pt*_total_*), *M* is the atomic weight of Pt (195.1 g/mol), *ρ_site_* is the density of the Pt surface site (12.5 Pt atoms/nm^2^), *d_av_* is the average particle size (nm), *ρ_metal_* is the metal density of Pt (21.45 g/cm^3^) and *N* is the Avogadro constant, giving *D* = 1.13/*d_av_* (*d_av_* in nm) for Pt.

**Calculation of the conversion and selectivity in the hydrogenation**

No else products but 3-MeCol, 3-MeBal, and 3-MeBol are detected by GC as reaction products under the experimental conditions used. The input carbon and output carbon are determined by sampling the reaction mixture before and after the reaction using the external standard method. The carbon balance values based on these products are near 100 %. The conversion and selectivity were calculated according to the following formulas:

$Conv._{3-MeCal}\%=\frac{A_{3-MeCol}+A_{3-MeBol}+A_{3-MeBal}}{A_{3-MeCal}+A_{3-MeCol}+A_{3-MeBol}+A_{3-MeBal}}\times100\%$,

$Select._{3-MeCol}\%=\frac{A_{3-MeCol}}{A_{3-MeCol}+A_{3-MeBol}+A_{3-MeBal}}\times100\%$,

$Select._{3-MeBol}\%=\frac{A_{3-MeBol}}{A_{3-MeCol}+A_{3-MeBol}+A_{3-MeBal}}\times100\%$,

$Select._{3-MeBal}\%=\frac{A_{3-MeBal}}{A_{3-MeCol}+A_{3-MeBol}+A_{3-MeBal}}\times100\%$,

**Determination of initial turn-over frequency (TOF)**

The initial turn-over frequency (TOF) was calculated based on the yield of 3-MeCol, according to the following equation:[^5^](#_ENREF_5)

$TOF=\frac{\frac{\rho V}{M_{3-Mecal}}\times Comp.\%}{\frac{m_{cat}\times L_{Pt}\%\times D_{Pt}\%}{M_{Pt}}\times t}$,

Where *ρ* is the density of 3-MeCal (0.878 g/cm^3^), *V* is the volume of 3-MeCal (2 mL), *M_3-MeCal_* is the molecular weight of 3-MeCal (84.12 g/mol), *Comp.*% is the composition of 3-MeCol in the reaction system at the time *t*, *m_cat_* is the mass of Pt/MCNT catalyst (0.1 g), *L_Pt_* is the Pt loading of Pt/MCNT catalyst (%), *M_Pt_* is the molar mass of Pt (195.08 g/mol), *D_Pt_* is the dispersion of Pt (%), and *t* is the reaction time (s).

**Determination of the specific reaction rate (SRR)**

For the Pt/Fe_3_O_4_-MCNT catalysts, TOF is hard to be calculated due to the difficulty to determine the Pt dispersion in the presence of ferrite. Consequently, the specific reaction rate (SRR) is used to describe the activity of Pt catalyst and calculated according to the following equation:

,

Where *m_catal_*. is the mass of the used catlayst (0.2 g), *ξ* is the reaction progress, *L_Pt_* is the Pt loading, *t* is the reaction time (min), *Conv.*% is the conversion of 3-MeCal, *V_3-MeCal_* is the reactant volume (2 mL), *ρ_3-MeCal_* is the density of the reactant (0.878 g/mL), and *M_3-MeCal_* is the molar mass of the reactant (84.12 g/mol).

**Hydrogenation results of the Pt/MCNT catalysts**

**Figure S1** 3-methylcrotonaldehyde hydrogenation results of the Pt/MCNT catalysts. (a) 0.5Pt/MCNT, (b) 3Pt/MCNT, and (c) 5Pt/MCNT. Constant H_2_ pressure of 1.4 MPa was used.

**Recycling test on 3Pt/MCNT**

Table S1 Recycling results of the hydrogenation of 3-MeCal over 3Pt/MCNT

| Cycle | Conversion / % | Selectivity / % | | |
| --- | --- | --- | --- | --- |
|  |  | 3-MeBal | 3-MeBol | 3-MeCol |
| 1 | 32.1 | 1.5 | 2.9 | 95.6 |
| 2 | 31.1 | 1.0 | 3.0 | 96.0 |
| 3 | 28.8 | 1.9 | 3.3 | 95.8 |
| 4 | 30.2 | 2.0 | 3.0 | 95.0 |
| 5 | 29.6 | 2.0 | 3.1 | 94.9 |

Reaction conditions: T=80 °C, t=20 min, catalyst amount: 0.1 g, 3-MeCal dose: 2 ml, ethanol dose: 16 ml, deionized water: 2 ml, initial H_2_ pressure: 1.4 MPa. No H_2_ was fed during the reaction.

**Recycling test on 3Pt/5Fe_3_O_4_-MCNT**

**Table S2** Recycling results of the hydrogenation of 3-MeCal over 3Pt/5Fe_3_O_4_-MCNT catalyst

| Cycle | Conversion/% | Selectivity/% | | |
| --- | --- | --- | --- | --- |
|  |  | 3-MeCol | 3-MeBal | 3-MeBol |
| 1 | 79.7 | 98.8 | 0.3 | 0.8 |
| 2 | 81.2 | 98.6 | 0.3 | 1.0 |
| 3 | 79.8 | 98.2 | 0.5 | 1.2 |
| 4 | 81.7 | 98.0 | 0.4 | 1.6 |
| 5 | 78.0 | 98.3 | 0.5 | 1.2 |

Reaction conditions: T=80 °C, t=3 h, catalyst amount: 0.1 g, 3-MeCal dose: 2 ml, ethanol dose: 16 ml, deionized water: 2 ml, initial H_2_ pressure: 1.4 Mpa. No H_2_ was fed during the reaction.

**Hydrogenation of 3-methyl crotonalcohol**

**Figure S2.** Hydrogenation of 3-methyl crotonalcohol over 3Pt/5Fe_3_O_4_-MCNT catalyst.

Reaction conditions: T=80 °C, H_2_ pressure: 0.8 MPa, catalyst amount: 0.1 g, 3-MeCol dose: 2 ml, ethanol dose: 16 ml, deionized water: 2 ml.

**References**

1. M. Brust, M. Walker, D. Bethell, D. J. Schiffrin and R. Whyman, *J. Chem. Soc., Chem. Commun.*, 1994, **0**, 801-802.

2. S. Sun and H. Zeng, *J. Am. Chem. Soc.*, 2002, **124**, 8204-8205.

3. R. Hao, R. Xing, Z. Xu, Y. Hou, S. Gao and S. Sun, *Adv. Mater.*, 2010, **22**, 2729-2742.

4. J. J. F. Scholten, A. P. Pijpers and M. L. Hustings, *Catal. Rev.*, 1985, **27**, 151-206.

5. S. Kozuch and J. M. L. Martin, *ACS Catalysis*, 2012, 2787-2794.
